# Clinical Impact of Multifocality and Bilaterality on Lymph Node Metastasis in Papillary Thyroid Microcarcinoma

**DOI:** 10.3390/diagnostics16020208

**Published:** 2026-01-09

**Authors:** Merima Goran, Marko Buta, Srdjan Nikolic, Nada Santrac, Nikola Jeftic, Nevena Savkovic, Jovan Raketic, Zoran Kozomara, Natasa Medic-Milijic, Ana Cvetkovic, Saska Pavlovic, Ivan Markovic

**Affiliations:** 1 School of Medicine, University of Belgrade, Dr Subotica 8, 11000 Belgrade, Serbia; 2 Surgical Oncology Clinic, Institute for Oncology and Radiology of Serbia, Pasterova 14, 11000 Belgrade, Serbia; 3 Department of Pathology, Institute for Oncology and Radiology of Serbia, Pasterova 14, 11000 Belgrade, Serbia; 4 Department of Anesthesiology, Institute for Oncology and Radiology of Serbia, Pasterova 14, 11000 Belgrade, Serbia

**Keywords:** papillary thyroid microcarcinoma, multifocality, bilaterality, lymph node metastases

## Abstract

**Objective:** Papillary thyroid microcarcinoma (PTMC) often presents with multifocality and bilaterality, but the clinical significance of these features and their association with cervical lymph node metastases (LNMs) remain debated. The aim of this study was to investigate the patterns of multifocality and bilaterality in PTMC and their association with central and lateral neck lymph node metastases. **Methods:** This retrospective study analyzed 254 patients with histologically confirmed PTMC treated at the Institute for Oncology and Radiology of Serbia between 2004 and 2016. All patients underwent total thyroidectomy with central and, when indicated, lateral neck dissection. Associations between multifocality, bilaterality, and cervical LNM were evaluated using appropriate statistical tests. A *p*-value < 0.05 was considered statistically significant. **Results:** Multifocal tumors were present in 40.55% of patients, with bilateral involvement in 27.17%. Cervical LNM occurred in 33.07% of patients, with 26.77% showing central and 20.08% lateral metastases. Patients with multifocal tumors were associated with significantly higher proportions of male patients (*p* = 0.0283), higher rates of capsular invasion (*p* = 0.0002), larger tumor size (*p* = 0.0134), and increased incidence of LNM (*p* = 0.0152). Bilateral tumors were associated with larger tumor size (*p* = 0.0004) and more frequent capsular invasion (*p* = 0.0248), but not with a statistically significant increase in LNM. The number of tumor foci was strongly associated with both central and lateral LNM (*p* < 0.001). **Conclusions:** Multifocality, particularly with a higher number of tumor foci, is significantly associated with more aggressive tumor features and higher rates of cervical lymph node metastases in PTMC. While bilaterality also reflects a more aggressive phenotype, it was not independently predictive of LNM. These findings underscore the importance of careful risk stratification in PTMC and suggest that multifocality should inform surgical and follow-up strategies.

## 1. Introduction

According to the World Health Organization (WHO), papillary thyroid microcarcinomas (PTMCs) are papillary thyroid carcinomas (PTCs) measuring ≤ 10 mm in greatest dimension [1]. It is estimated that PTMCs comprise up to 50% of all PTCs. The rising incidence of these tumors likely reflects improved ultrasound diagnostics, expanded surgical indications, and more detailed histopathological processing using thinner tissue sections [2,3]. Despite the increasing incidence, overall mortality from PTMC has remained stable, as these tumors are typically indolent and associated with an excellent prognosis [4].

Multifocality is a common feature of PTMC with its etiology remaining insufficiently understood. Some studies suggest multicentric malignant transformation of follicular cells, supported by evidence of distinct clonal origins among multiple tumor foci [5,6]. Other studies suggest intraglandular spread of a primary tumor through the thyroid’s rich lymphatic network, supported by evidence of identical clonal origin among tumor foci [7]. Bansal et al. found that unilateral multifocal tumors often share the same mutation, while bilateral tumors typically show different mutational profiles [8]. Some authors therefore consider unilateral multifocal and bilateral tumors to be separate entities, with differing clinical behavior, biology, and outcomes, as supported by their divergent mutation statuses [9].

Lymph node metastases (LNMs) indicate regional dissemination of disease to the cervical lymph nodes. This most commonly involves the central neck compartment, level VI, but may also affect the lateral compartments, including levels II through V [10]. Their presence indicates more aggressive tumor behavior, particularly when evident preoperatively, when metastases are larger, or when multiple lymph nodes are involved [11]. During the 2000s, the optimal extent of surgery for PTMC was still under discussion. Many tertiary centers performed total thyroidectomy with prophylactic central neck dissection (CND) to improve pathological staging and guide adjuvant therapy, particularly in multifocal or bilateral disease. Sentinel lymph node biopsy (SLNB) was also explored as a technique to detect occult lateral neck metastases in clinically node-negative patients, enabling a more accurate assessment of disease spread and potential aggressiveness. Although these procedures were not universally adopted, several studies have demonstrated their diagnostic and prognostic value in refining surgical planning and risk stratification for PTMC [12,13,14,15]. However, despite numerous studies addressing the clinicopathological features of PTMC, data on the association between multifocality, bilaterality, and the pattern of cervical lymph node involvement, particularly in cohorts where both central and lateral neck regions were surgically assessed through standardized procedures such as central neck dissection and sentinel lymph node biopsy, remain limited. Further clarification of these relationships may contribute to a better understanding of disease behavior and support future refinements in risk stratification and management strategies.

The aim of this study was to investigate multifocality and bilaterality patterns in PTMCs and their association with the presence of cervical LNMs.

## 2. Materials and Methods

### 2.1. Patients and Data

This retrospective study included patients treated at the Institute for Oncology and Radiology of Serbia (IORS) between January 2004 and December 2016. The study included all patients with histopathologically confirmed PTMC and who underwent surgical evaluation of both central and lateral neck lymph nodes. Patients were excluded if they had tumors larger than 10 mm or incomplete lymph node assessment (only central, only lateral or none). Routine preoperative evaluation for all patients included neck and abdominal ultrasound, chest and neck X-rays (to assess tracheal deviation or compression), laryngoscopy to assess vocal cord function, thyroid function testing, and anesthesiologic assessment. Treatment decisions were made by a multidisciplinary thyroid tumor board. Preoperative, operative, and follow-up data were extracted from the institutional database.

The study was conducted in accordance with the Declaration of Helsinki and approved by the Ethics Committee of the Institute for Oncology and Radiology of Serbia (protocol code 01-1/2025/3280; 20 October 2025). The study was conducted using de-identified clinical data collected during routine care, with no patient contact or access to identifiable information. In accordance with institutional and national regulations, the ethics committee granted a waiver of informed consent.

### 2.2. Treatment

The treatment strategy for patients with preoperatively cytologically or intraoperatively frozen section–proven PTMC involved total or near-total thyroidectomy. In all patients without clinical evidence of central lymph node metastasis, prophylactic CND was performed, whereas those with clinically positive central lymph nodes underwent therapeutic CND. Sentinel lymph node biopsy (SLNB) was performed in patients without clinical or radiological evidence of lateral cervical lymph node metastases (cN0). The procedure was carried out on the affected side, or bilaterally in cases of bilateral PTMC, following the technique described by Džodić et al. [16]. After thyroid exposure and before gland mobilization, approximately 0.2 mL of 1% methylene blue dye was injected peritumorally using a fine needle to identify stained lymphatic vessels and sentinel nodes. The excised sentinel lymph nodes were sent for intraoperative frozen section analysis, and the results guided further surgical management. In cN0 patients with negative SLNB no additional lateral neck dissection was undertaken and patients were subjected to standard follow-up. If lateral lymph node metastases were clinically evident (cN1b) or confirmed by sentinel lymph node biopsy, a modified radical neck dissection (MRND) of the affected side was performed during the same procedure. In most patients who underwent MRND, it included lymph node levels II through IV, while level V was dissected only in cases with clinically or intraoperatively evident metastases. The spinal accessory nerve, internal jugular vein, and sternocleidomastoid muscle were preserved in all cases.

All cases were histopathologically confirmed as PTMC following standard processing of thyroidectomy specimens. Both thyroid lobes were examined in detail to assess tumor focality, bilaterality, and capsular invasion. All tissue samples, including thyroid, CND, SLNB, and MRND specimens, were examined by experienced endocrine pathologists using standardized protocols to determine the presence and extent of lymph node metastases.

### 2.3. Statistical Analysis

Categorical variables were described using frequencies (percentages), while mean, median, standard deviation (SD), and range were used for numeric variables. Multifocality and bilaterality were analyzed in relation to patient demographics, tumor characteristics, and the presence of cervical LNMs in order to assess potential associations. The normality of distribution for continuous variables was assessed using visual methods (Normal Q-Q plots and histograms) and statistical tests (Kolmogorov–Smirnov and Shapiro–Wilk tests). To compare differences between groups, the Pearson’s χ^2^ test, Fisher’s exact test, or the Wilcoxon rank-sum test were applied, depending on the type and distribution of the variables. A *p*-value of <0.05 was considered statistically significant. In cases of multiple testing on the same dataset, Bonferroni correction was applied to adjust the significance threshold. Statistical analyses were performed using R version 3.3.2 (2016-10-31)—“Sincere Pumpkin Patch”; Copyright (C) 2016 The R Foundation for Statistical Computing; Platform: x86_64-w64-mingw32/x64 (64-bit) (available at: www.r-project.org; downloaded: 21 January 2017).

## 3. Results

**Figure 1:**
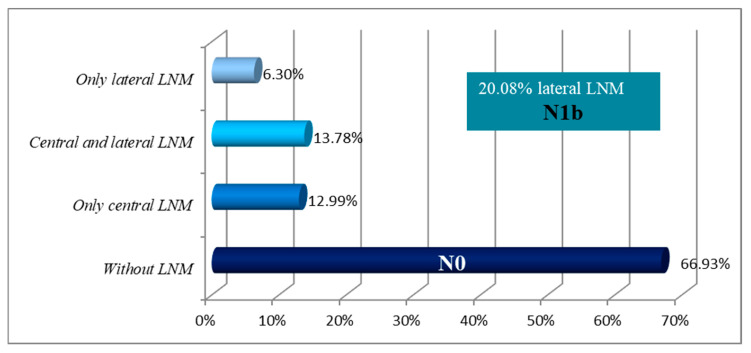
Percentage of LNMs by neck regions.

A total of 254 patients met the inclusion criteria for this study. The study population included a predominance of female patients (84.65%). Average age of patients was 47.7 years (Table 1). Metastases in the cervical lymph nodes were histopathologically confirmed in 84 (33.07%) patients. Metastases limited to the central neck compartment (N1a) were found in 33 patients (12.99%). Skip metastases, involving only the lateral compartment without central lymph node involvement, were identified in 16 patients (6.30%), while both central and lateral metastases were present in 35 patients (13.78%). These two groups were collectively classified as N1b (Table 1, Figure 1). Multifocal disease was present in 103 patients (40.55%). Notably, among patients with multifocal tumors, the number of those with bilateral involvement was 69 (66.99%) compared with 34 (33.01%) in those with unilateral multifocal disease. Capsular invasion was detected in 20.87% of patients, and vascular invasion was observed in only one patient (Table 2).

The association between the number of PTMC foci and patient and tumor characteristics is presented in Table 3. A statistically significant difference in sex distribution was observed between patients with unifocal and multifocal tumors, with the proportion of male patients being 11.26% in unifocal group compared to 21.36% in the multifocal group. Multifocal tumors were also associated with larger average tumor size compared to unifocal tumors. Additionally, the prevalence of capsular invasion and the incidence of cervical lymph node metastases (central, lateral, or both) were significantly higher among patients with multifocal tumors.

The association between the number of PTMC foci and the presence of cervical LNM is presented in Table 4. Patients with cervical LNM (central, lateral, or both) had, on average, a greater number of PTMC foci compared to those without metastases. Furthermore, an increasing number of PTMC foci were associated with a higher likelihood of central and overall LNM. A similar trend was observed for lateral LNM, although it did not reach statistical significance.

The association between laterality and patient and tumor characteristics in subgroup of patients with multifocal PTMC is presented in Table 5. Patients with bilateral tumors had larger average tumor size and a greater number of tumor foci compared to those with unilateral multifocal disease. Capsular invasion was also more frequently observed in the bilateral group. No significant difference was observed in the presence of LNM between patients with unilateral and bilateral multifocal PTMC.

Two patients experienced recurrent laryngeal nerve (RLN) injury during surgery. In one case, the RLN was accidentally transected intraoperatively, promptly recognized, and repaired with direct perineurial suturing. The second patient developed transient RLN palsy, which resolved spontaneously within six months. No cases of permanent hypoparathyroidism or other major thyroid or neck dissection-related complications were reported. Local recurrence occurred in two female patients and distant recurrence occurring in one male patient with occult PTMC diagnosed via biopsy of an enlarged cervical lymph node.

## 4. Discussion

The reported prevalence of multifocality in papillary thyroid carcinomas varies widely, ranging from 18% to 87.5%, largely due to differences in histopathological tissue processing [4,17]. In our study, multifocal tumors were identified in 40.55% of patients, consistent with findings from other studies [18,19]. The prevalence of bilaterality in PTMC typically ranges from 10–30%, though rates up to 68.1% have been reported, largely reflecting differences in histopathological techniques [18,20]. In our cohort, bilateral tumors were present in 27% of patients, while 13% showed unilateral multifocality. Comparable rates of bilaterality were observed by Varshney et al. (28.8%) and Karatzas et al. (24.1%) [18,19].

The mean number of tumor foci per patient was 1.97 in our cohort, compared with 1.6 in a similar series. In multifocal tumors, secondary foci were generally smaller than the dominant lesion, averaging 1.8 mm in our study versus 3.2 mm reported by So YK et al. [21]. In our study, total number of tumor foci was significantly higher in patients with LNM compared to those without (2.79 vs. 1.56, *p* = 0.0009). Similarly, Al Afif reported an association between tumor focus number and central LNM in PTC [22]. In the study by Lee HS et al., the incidence of LNM in multifocal PTMC ≤ 5 mm was 42.3%, and multifocality was identified as the sole predictor of LNM in small PTMC [23].

Tumor size is another factor linked to increased aggressiveness, with larger tumors being more likely to metastasize to lymph nodes. In PTMC, tumors > 5 mm are considered more aggressive due to a higher risk of LNM [24,25]. In our study, multifocal tumors were significantly larger than solitary tumors (5.17 mm vs. 4.8 mm, *p* = 0.0134). Multifocality was more common in tumors measuring 6–10 mm compared to those ≤ 5 mm, although without statistical significance, consistent with findings by So YK et al. [21], while Kaliszewski and Kim reported statistically significant associations [26,27]. Bilaterality was also more common in larger tumors, consistent with findings by Kaliszewski [26]. In our study, capsular invasion was more frequent in multifocal and bilateral tumors. Karatzas et al. and Cupisti reported associations between multifocality, bilaterality, and capsular invasion, consistent with our findings [18,28].

LNMs were present in 33.07% of patients, in our study, with central compartment metastases identified in 26.77% and lateral metastases in 20.08%. Similar results were reported by Cheng F and Luo Y, but with the frequency of lateral LNMs being notably higher in our cohort [29,30]. LNMs occurred more frequently in multifocal tumors, supporting the view that multifocality reflects more aggressive behavior. Although bilateral tumors also showed a trend toward increased LNMs, the difference was not statistically significant. Similarly, Zheng et al. found a strong association between multifocality and central LNM, but not with bilaterality [31]. However, some studies have reported no independent association between multifocality and central LNM in multivariate analyses [32,33].

A meta-analysis by Pyo et al. showed significantly higher recurrence rate in multifocal tumors [34], while Kuo et al. reported worse survival in multifocal compared to solitary tumors, although no difference was found between multifocal PTMC and larger multifocal carcinomas [35]. Incomplete surgical treatment is a recognized cause of recurrence in multifocal disease, supporting total thyroidectomy as the optimal approach for PTC, including PTMC, particularly when multifocality is confirmed preoperatively. In our cases, recurrence likely resulted from additional aggressive tumor features or incomplete lateral neck dissection (levels II–V), as both recurrences arose in previously dissected regions. Kaliszewski reported a higher incidence of distant metastases in multifocal and bilateral PTMC, with rates of 0.9% in solitary, 14.6% in multifocal, and 13.3% in bilateral tumors [26]. In contrast, only one patient in our study developed distant metastases, originating from an occult solitary PTMC.

In recent years, active surveillance has emerged as a viable and evidence-based management option for carefully selected patients with low-risk PTMC. Large prospective and multicenter studies have demonstrated that, in the absence of high-risk features, active surveillance can safely replace immediate surgery without compromising oncologic outcomes [36,37,38]. Adequate patient selection remains the cornerstone of this approach, requiring careful assessment of clinical, radiological, and pathological factors that may predict disease progression. Recent reviews have emphasized that features such as multifocality, extrathyroidal extension, and lymph node metastases should prompt caution when considering active surveillance [39,40]. The results of our study contribute to this evolving body of evidence by identifying multifocality as a marker associated with more aggressive disease behavior and an increased risk of nodal metastasis, suggesting that this feature can be incorporated into comprehensive decision-making algorithms when selecting patients for active surveillance. Although bilaterality was not significantly associated with lymph node metastasis in our cohort, it correlated with other markers of aggressiveness, including larger tumor size, capsular invasion, and multifocality. Therefore, bilaterality may also represent a relative risk factor when evaluating patients for active surveillance, and its presence should encourage a more individualized, cautious approach

This study comes with several limitations inherent to its retrospective design. The analysis was based on data collected from a single tertiary institution, which may limit the generalizability of the findings to broader populations. While efforts were made to ensure data accuracy, the retrospective nature introduces potential for selection and information bias. Molecular profiling was not performed, precluding evaluation of potential genetic differences between multifocal and bilateral tumors. Additionally, multivariate analysis was not conducted, limiting the ability to control for confounding variables This approach was not pursued due to the interrelation among several key variables, such as multifocality, bilaterality, and number of tumor foci, which could introduce collinearity and compromise model stability and interpretability; moreover, such analysis would require a larger, prospectively designed study specifically powered for predictive modeling. The absence of long-term outcome analysis limits conclusions regarding recurrence and overall survival.

## 5. Conclusions

Multifocality in PTMC is a common finding and was significantly associated with male sex, larger tumor size, capsular invasion, and higher rates of lymph node metastases. Bilateral tumors also showed more aggressive features but without a statistically significant association with lymph node involvement. Although local recurrence was rare, it occurred exclusively in patients with multifocal disease, suggesting a trend toward more aggressive behavior. While active surveillance remains appropriate for carefully selected low-risk patients, the presence of multifocality, especially when combined with other high-risk features, should prompt a more cautious and individualized approach. These findings indicate that multifocal PTMC may represent a biologically distinct subtype, although confirmation requires studies incorporating long-term survival data and molecular profiling (BRAF, RAS, TERT, RET/PTC). Further research should define the independent prognostic role of multifocality and its potential inclusion in future risk stratification models for PTMC.

## Figures and Tables

**Table 1:** Patient characteristics.

Patient Characteristics		No (%)
Sex	Male	39 (15.35)
	Female	215 (84.65)
Age (years)	Mean (SD)	47.75 (12.42)
	Median (Range)	49 (23–75)
Age categories (years)	<55	162 (63.78)
	≥55	92 (36.22)
LNM	Yes	84 (33.07)
	No	170 (66.93)
CLNM	Yes	68 (26.77)
	No	186 (73.23)
LLNM	Yes	51 (20.08)
	No	203 (79.92)
TLNMN	Mean (SD)	4.94 (5.24)
	Median (Range)	3 (1–25)
Total		254 (100)

LNMs—lymph node metastases; CLNMs—central lymph node metastases; LLNMs—lateral lymph node metastases; TLNMN—total lymph node metastases number; SD—standard deviation.

**Table 2:** Tumor characteristics.

Tumor Characteristics		No (%)
Tumor size (mm)	Mean (SD)	5.17 (2.99)
	Median (Range)	5 (0.5–10)
Tumor size category (mm)	≤5	149 (58.66)
	6–10	105 (41.34)
Number of foci	Unifocal	151 (59.45)
	Multifocal	103 (40.55)
Average number of foci	Mean (SD)	1.97 (2.14)
	Median (Range)	1 (1–20)
Average number of foci (multifocal tumors)	Mean (SD)	3.39 (2.82)
	Median (Range)	2 (2–20)
Smallest focus size (multifocal tumors) (mm)	Mean (SD)	1.8 (1.47)
	Median (Range)	1.5 (0.4–8)
Multifocal tumor laterality	Unilateral	34 (33.01)
	Bilateral	69 (66.99)
Capsular invasion	Yes	53 (20.87)
	No	200 (78.74)
	No data	1 (0.39)
Vascular invasion	Yes	1 (0.39)
	No	253 (99.61)
Thyroiditis	Yes	101 (39.76)
	No	153 (60.24)
Total		254 (100)

SD—standard deviation.

**Table 3:** Association between the number of PTMC foci and patient and tumor characteristics.

Characteristic		Number of Foci		Test (Pearson χ^2^)
		Unifocal No (%)	Multifocal No (%)	
Sex	Male	17 (11.26)	22 (21.36)	*p* = 0.0283
	Female	134 (88.74)	81 (78.64)	
Age (years)	Mean (SD)	47.57 (12.82)	48.01 (11.86)	*p* = 0.7840 ^♦^
	Median (Range)	49 (23–75)	50 (25–73)	
Age category (years)	<55	96 (63.58)	66 (64.08)	*p* = 0.9349
	≥55	55 (36.42)	37 (35.92)	
Tumor size (mm)	Mean (SD)	4.8 (3)	5.71 (2.9)	*p* = 0.0134 ^♦^
	Median (Range)	4 (0.5–10)	5 (0.5–10)	
Tumor size category (mm)	≤5	95 (62.91)	54 (52.43)	*p* = 0.0956
	6–10	56 (37.09)	49 (47.57)	
Capsular invasion	Yes	20 (13.25)	33 (32.04)	*p* = 0.0002
	No	131 (86.75)	69 (66.99)	
	No data	0 (0)	1 (0.97)	
Vascular invasion	Yes	0 (0)	1 (0.97)	*p* = 0.4055 *
	No	151 (100)	102 (99.03)	
Thyroiditis	Yes	59 (39.07)	42 (40.78)	*p* = 0.7853
	No	92 (60.93)	61 (59.22)	
LNM	Yes	41 (27.15)	43 (41.75)	*p* = 0.0152
	No	110 (72.85)	60 (58.25)	
CLNM	Yes	31 (20.53)	37 (35.92)	*p* = 0.0065
	No	120 (79.47)	66 (64.08)	
LLNM	Yes	23 (15.23)	28 (27.18)	*p* = 0.0196
	No	128 (84.77)	75 (72.82)	
Total		151 (100)	103 (100)	-

LNMs—lymph node metastases; CLNMs—central lymph node metastases; LLNMs—lateral lymph node metastases; SD—standard deviation; ♦ Wilcoxon rank sum; * Fisher’s Exact Test; No—number of patients.

**Table 4:** Association between the number of PTMC foci and the presence of neck lymph node metastases.

	No-LNM No (%)	LNM No (%)	Test Wilcoxon Rank Sum
Number of foci			
Mean (SD)	1.56 (0.95)	2.79 (3.34)	*p* = 0.0009
Median (Range)	1 (1–6)	2 (1–20)	
Total patients	170	84	
Number of foci unilaterally			
Mean (SD)	1.92 (0.91)	3.46 (3.63)	*p* = 0.0050
Median (Range)	2 (1–5)	2 (1–19)	
1 focus	22 (36.67)	9 (20.93)	*p* = 0.0398 ^⁂^
2 foci	25 (40.67)	15 (34.88)	
≥3 foci	13 (21.67)	19 (44.19)	
Total patients	60	43	
	**No-CLNM** **No (%)**	**CLNM** **No (%)**	**Test** **Wilcoxon rank sum**
Number of foci			
Mean (SD)	1.56 (0.93)	3.09 (3.63)	*p* = 0.0001
Median (Range)	1 (1–6)	2 (1–20)	
Total patients	186	68	
Number of foci unilaterally			
Mean (SD)	1.89 (0.9)	3.76 (3.83)	*p* = 0.0005
Median (Range)	2 (1–5)	2 (1–19)	
1 focus	25 (37.88)	6 (16.22)	*p* = 0.0080 ^⁂^
2 foci	27 (40.91)	13 (35.14)	
≥3 foci	14 (21.21)	18 (48.65)	
Total patients	66	37	
	**No-LLNM** **No (%)**	**LLNM** **No (%)**	**Test** **Wilcoxon rank sum**
Number of foci			
Mean (SD)	1.66 (1.13)	3.2 (4.01)	*p* = 0.0021
Median (Range)	1 (1–8)	2 (1–20)	
Total patients	203	51	
Number of foci unilaterally			
Mean (SD)	2.04 (1.07)	3.96 (4.31)	*p* = 0.0099
Median (Range)	2 (1–6)	2 (1–19)	
1 focus	26 (34.67)	5 (17.86)	*p* = 0.0850 ^⁂^
2 foci	30 (40)	10 (35.71)	
≥3 foci	19 (25.33)	13 (46.43)	
Total patients	75	28	

LNMs—lymph node metastases; CLNMs—central lymph node metastases; LLNMs—lateral lymph node metastases; SD—standard deviation; No—number of patients; ⁂—Pearson’s χ^2^ test.

**Table 5:** Association between the multifocal PTMC laterality and patient and tumor characteristics.

Characteristic		Multifocal Tumor Laterality		Test (Pearson χ^2^)
		Unilateral No (%)	Bilateral No (%)	
Sex	Male	7 (20.59)	15 (21.74)	*p* = 0.8934
	Female	27 (79.41)	54 (78.26)	
Age (years)	Mean (SD)	50.62 (11.07)	46.72 (12.1)	*p* = 0.1584 ^♦^
	Median (Range)	52.5 (31–73)	46 (25–69)	
Age category (years)	<55	19 (55.88)	47 (68.12)	*p* = 0.2236
	≥55	15 (44.12)	22 (31.88)	
Tumor size (mm)	Mean (SD)	4.22 (2.57)	6.45 (2.78)	*p* = 0.0004 ^♦^
	Median (Range)	4.5 (0.5–10)	6 (1.5–10)	
Tumor size category (mm)	≤5	27 (79.41)	27 (39.13)	*p* = 0.0001
	6–10	7 (20.59)	42 (60.87)	
Capsular invasion	Yes	6 (17.65)	27 (39.13)	*p* = 0.0248
	No	28 (82.35)	41 (59.42)	
	No data	0 (0)	1 (1.45)	
Vascular invasion	Yes	1 (2.94)	0 (0)	*p* = 0.3301 *
	No	33 (97.06)	69 (100)	
Thyroiditis	Yes	15 (44.12)	27 (39.13)	*p* = 0.6281
	No	19 (55.88)	42 (60.87)	
Total number of foci	Mean (SD)	2.71 (1.34)	3.73 (3.27)	*p* = 0.0380 ^♦^
	Median (Range)	2 (2–8)	3 (2–20)	
LNM	Yes	12 (35.29)	31 (44.93)	*p* = 0.3512
	No	22 (64.71)	38 (55.07)	
CLNM	Yes	10 (29.41)	27 (39.13)	*p* = 0.3337
	No	24 (70.59)	42 (60.87)	
LLNM	Yes	8 (23.53)	20 (28.99)	*p* = 0.5584
	No	26 (76.47)	49 (71.01)	
Total		34 (100)	69 (100)	-

LNMs—lymph node metastases; CLNMs—central lymph node metastases; LLNMs—lateral lymph node metastases; SD—standard deviation; No—number of patients; ♦ Wilcoxon rank sum test; *—Fisher’s Exact Test.

## Data Availability

To protect the privacy and confidentiality of patients in this study, clinical data have not been made publicly available in a repository of the article; however, they will be made available upon reasonable request to the corresponding author.

## Abbreviations

The following abbreviations are used in this manuscript:

PTMC	papillary thyroid microcarcinomas
PTC	papillary thyroid carcinomas
LNMs	lymph node metastases
IORS	Institute for Oncology and Radiology of Serbia
SLNB	sentinel lymph node biopsy
MRND	modified radical neck dissection
CLNMs	central lymph node metastases
LLNMs	lateral lymph node metastases
TLNMN	total lymph node metastases number
SD	standard deviation
RLN	recurrent laryngeal nerve
